# Circadian Rhythms and Depression in Adolescents: A Comparative Analysis of First Episode and Recurrent-Episode Groups

**DOI:** 10.3390/jpm13121665

**Published:** 2023-11-28

**Authors:** Young-Soo Jang, Hye-Mi Cho, Young-Eun Mok, Su-Hyuk Chi, Chang-Su Han, Moon-Soo Lee

**Affiliations:** Department of Psychiatry, Korea University Guro Hospital, Seoul 08308, Republic of Korea; zerg0506@korea.ac.kr (Y.-S.J.); vosej28@korea.ac.kr (H.-M.C.); youngeuncee@korea.ac.kr (Y.-E.M.); link0710@korea.ac.kr (S.-H.C.); hancs@korea.ac.kr (C.-S.H.)

**Keywords:** circadian rhythm, adolescent, depressive disorder, chronicity

## Abstract

Circadian rhythm disruptions are a hallmark feature of mood disorders. Patients experiencing acute depressive episodes report noticeable changes in their sleep–wake cycles. This research explains the association between depression and various circadian rhythm metrics, explicitly focusing on adolescents diagnosed with depressive disorders. Adolescence is a critical period marked by significant physiological and psychological changes, making it imperative to understand how mood disorders manifest during this phase. However, there have been minimal specific studies in pediatric populations to determine whether circadian rhythm changes differ between adolescents with first and multiple-recurrent depressive episodes. Our study involved a group of 61 adolescents aged between 13 and 18. We performed a cross-sectional study of a clinical population of patients presenting to a child and adolescent psychiatry clinic diagnosed with depression. Participants were asked to complete self-report evaluations using several tools: the Korean version of the Biological Rhythms Interview of Assessment in Neuropsychiatry (K-BRIAN), the Korean Translation of Composite Scale to Measure Morningness-Eveningness (KtCS), and the Seasonal Pattern Assessment Questionnaire (SPAQ). Tools such as the Children’s Depression Inventory (CDI), State-Trait Anxiety Inventory (STAI), and K-Mood Disorder Questionnaire (K-MDQ) were employed for the assessment of clinical characteristics of depression. Based on the frequency of their depressive episodes, participants were bifurcated into two distinct groups: those experiencing their first episode (n = 22, mean age: 15.09 ± 1.44 years) and those with recurrent episodes (n = 39, mean age: 15.95 ± 1.26 years). At first, the two groups’ data revealed no significant differences regarding mood or circadian rhythm metrics (CDI: first episode 26.18 ± 10.54 and recurrent episode 25.90 ± 10.59, STAI-S: first episode 56.91 ± 12.12 and recurrent episode 57.49 ± 11.93, STAI-T: first episode 60.36 ± 11.63 and recurrent episode 59.09 ± 12.10, SPAQ-total: first episode 6.59 ± 4.86 and recurrent episode 6.77 ± 5.23, KtCS: first episode 30.32 ± 5.83 and recurrent episode 28.13 ± 7.36). However, we observed significant correlations between circadian rhythm disruptions and depression scales (CDI with SPAQ-weight (r = 0.26), KtCS (r = −0.48), K-BRIAN-sleep (r = 0.58), K-BRIAN-activity (r = 0.64), K-BRIAN-social (r = 0.71), and K-BRIAN-eating (r = 0.40)). These correlations were especially pronounced in the recurrent episode group, suggesting that with the progression and chronicity of depression, the relationship between circadian rhythms and depression becomes more intertwined and evident. In conclusion, especially in adolescents, as the severity and chronicity of depression increase, the interplay between circadian rhythms and mood disorders becomes more pronounced, warranting further research and clinical attention.

## 1. Introduction

Major depressive disorder (MDD) is a severe mental health condition that has garnered significant attention in recent years due to its profound impact on global health. It is also the predominant cause of disability globally among adolescents and young adults, with a marked surge in depressive symptoms from adolescence to early adulthood [1]. The global point prevalence of MDD, adjusting for methodological differences, was 4.7%. The prevalence of MDD and its debilitating effects underscore the urgency of understanding its underlying mechanisms and developing effective therapeutic interventions [2].

The intricate relationship between the environment and biological systems is evident in the daily light–dark cycle that predominantly dictates the behavioral and physiological rhythms observed across species. This natural rhythm, which has evolved over millennia, is crucial in regulating various physiological processes, including sleep, metabolism, and mood. The cycle is governed by the biological clock situated in the suprachiasmatic nucleus (SCN) of the anterior hypothalamus in mammals [3]. A growing body of evidence suggests a significant correlation between mood disorders, particularly MDD, and circadian rhythm disruptions. Sleep disturbances, which range from insomnia to hypersomnia, are not only hallmark symptoms of depression but are also integral to the diagnostic criteria for MDD [4]. It is alarming to note that a staggering 90% of patients undergoing acute depressive episodes report alterations in sleep–wake cycles [5]. While typical depression often manifests as early morning awakenings and fragmented sleep, atypical depression presents with extended sleep durations and delayed sleep times. However, both forms are accompanied by daytime fatigue, which can severely impact daily functioning. The association of both hypersomnia and insomnia with heightened suicidality underscores the imperative of addressing sleep disturbances in the context of depression [6].

Various therapeutic interventions have been developed and implemented to address these circadian rhythm disturbances. These include light and dark therapy, agomelatine [7], social rhythm therapy [8], and sleep phase advancement [3] to stabilize mood symptoms. These interventions’ effectiveness also highlights circadian rhythms’ crucial role in mood regulation.

While the diagnostic criteria for MDD remain consistent across adults, children, and adolescents, distinct differences in etiology, treatment response, and genetic makeup exist between these groups [9]. Adolescents with depression predominantly display vegetative symptoms like appetite and weight fluctuations, fatigue, and insomnia. In contrast, adults are more prone to anhedonia, diminished interest, and concentration challenges [10,11]. The recurrent nature of depression, with each subsequent episode often escalating in severity, especially among adolescents [12], further complicates the clinical picture. This pattern exacerbates the functional decline of affected individuals, imposing a substantial socio-economic burden. Given the propensity for adolescent-onset depression to become more chronic, there is an urgent need for proactive treatment and a deeper understanding of its pathogenesis. However, exploring circadian rhythms in pediatric and adolescent depression remains limited.

The intricate relationship between altered circadian rhythms and depression, especially in adolescents, warrants further investigation. Given the heightened risk of chronicity in adolescent depression, it is pivotal to probe the interplay between depression’s chronicity and circadian rhythms.

In the broader context, the increasing prevalence of MDD, especially among the younger population, is a pressing global health concern. The World Health Organization has identified depression as a leading cause of disability worldwide, emphasizing the need for comprehensive research and targeted interventions. The role of circadian rhythms in the onset and progression of MDD offers a promising avenue for therapeutic advancements. By delving deeper into the molecular and genetic underpinnings of circadian disruptions in MDD, researchers can pave the way for innovative treatments that align with personalized medicine principles. Furthermore, understanding the unique challenges adolescents face with MDD can inform early intervention strategies, potentially mitigating the long-term impacts of the disorder.

Interestingly, while depressed adults often exhibit altered circadian patterns, emerging research indicates that this phenomenon might extend to adolescents with depression. Compared to their peers, adolescents with depression experience exacerbated insomnia, reduced sleep duration, increased social jetlag, diminished activity, and heightened exposure to nocturnal artificial light [10]. This observation raises critical questions about the developmental aspects of MDD and the potential differences in its manifestation across different age groups. Contrary to adult patterns, we hypothesize that in adolescents, the influence of chronicity on circadian rhythms might be muted due to the relatively shorter illness duration. Understanding these nuances will be crucial in tailoring treatments to individual needs as personalized medicine evolves.

## 2. Materials and Methods

### 2.1. Participants

Adolescent outpatients aged 13–18 years diagnosed with major depressive disorder (MDD) per the DSM-5 criteria were recruited from the Department of Psychiatry at Korea University Guro Hospital in November 2020 for one year. All patients were referred to the child and adolescent psychiatry department at the general hospital for treatment of depression, where they were seen by trained child and adolescent psychiatrists and diagnosed with major depression. Eligibility criteria included the absence of cognitive impairment and the ability to provide written informed consent. All participants were divided into first-time and recurrent episode groups. For the recurrent group, we used the diagnosis when an individual has experienced two or more major depressive episodes, with an interval of at least two months symptom-free between episodes. Exclusions were made for patients at a high risk of suicide or self-harm. Both participants and their guardians were thoroughly briefed about the study, and informed consent was obtained. As this study is primarily concerned with depression, we excluded patients from the study if other major axis I psychiatric problems (e.g., other bipolar disorders, schizophrenia, and anxiety disorders) were included as co-morbidities. Due to the pediatric age group, benzodiazepines were not used, and mainly SSRI antidepressants—escitalopram, sertraline, and fluoxetine—that are acceptable for pediatric use were used, and additionally melatonin, trazodone, and in some cases, small amounts of quetiapine for sleep.

### 2.2. Assessments

#### 2.2.1. Depression

The Children’s Depression Inventory (CDI), a self-report scale by Kovacs and Beck (1977), evaluates childhood depression symptoms across five subdomains. The Korean version, adapted in 1990, was employed [13,14].

#### 2.2.2. Anxiety

The State-Trait Anxiety Inventory (STAI) by Spielberger (1970) assesses both state and trait anxiety. The Korean adaptation from 1989 was utilized in this study [15].

#### 2.2.3. Bipolarity

The Mood Disorder Questionnaire (MDQ) is a screening tool for potential bipolar disorder. The Mood Disorder Questionnaire for Adolescents (MDQ-A) is a widely recognized instrument tailored to identify symptoms of bipolar disorders in adolescent populations. The Korean adaptation, termed K-MDQ-A, has been translated and validated to ensure its relevance and accuracy for Korean adolescents. The K-MDQ-A consists of questions that probe into the occurrence and severity of mood-related symptoms, particularly those indicative of bipolar spectrum disorders. Respondents are instructed to reflect on their feelings and behaviors over a specific timeframe and select responses that most accurately depict their experiences. The questionnaire is divided into distinct sections, each focusing on different aspects of mood disturbances, such as manic/hypomanic episodes and the overall impact of these symptoms on daily functioning. A cumulative score is derived from the responses, with specific cut-off points aiding in the potential identification of bipolar disorders. In the Korean context, the K-MDQ-A has shown robust reliability and validity. Its sensitivity and specificity make it an invaluable tool for research and clinical settings, especially when working with Korean adolescents suspected of having mood disorders. The Korean version for adolescents (K-MDQ-A) was adopted [16,17].

#### 2.2.4. Seasonality

The Seasonal Pattern Assessment Questionnaire (SPAQ) is a self-report questionnaire developed by Dr. Norman E. Rosenthal and colleagues in the early 1980s as a tool to assess seasonal variations in mood and behavior. It was initially designed to identify individuals with Seasonal Affective Disorder (SAD), a type of depression that occurs at specific times of the year, typically during the fall and winter months when daylight is reduced. The SPAQ consists of several sections that evaluate various aspects of seasonal patterns:

Degree of Seasonality: Participants are asked to rate the extent of seasonal changes in sleep length, social activity, mood, weight, appetite, and energy level on a 5-point scale ranging from none to highly marked. The sum of these scores provides a “Global Seasonality Score” (GSS), which quantifies the overall degree of seasonality experienced by the individual.

Pattern of Seasonality: This section identifies the specific months in which the participant experiences the onset and offset of symptoms, allowing for determining the seasonal pattern of mood disturbances.

Impact of the Seasonal Pattern: Participants rate the impact of seasonal changes on their daily life, ranging from no problem to a severe problem that requires hospitalization.

Sleep and Weight Questions: These questions delve deeper into the participant’s sleep patterns and weight fluctuations across different seasons [18].

#### 2.2.5. Morningness-Eveningness

The Korean Translation of Composite Scale (KtCS), a localized version of the Morningness-Eveningness Questionnaire (MEQ) by Horne and Östberg (1976), gauges an individual’s peak alertness period [19,20]. The MEQ determines an individual’s chronotype—either a morning (M-type), evening (E-type), or neither (N-type)—based on their natural activity and sleep preferences through 19 questions, with scores ranging from 16 to 86. Commonly, M-type scores are 59–86, N-type 42–58, and E-type 16–41. This study utilized the KtCS, a Korean version of the MEQ.

#### 2.2.6. Biological Rhythm

The Biological Rhythms Interview of Assessment in Neuropsychiatry (BRIAN) is a validated instrument designed to assess disturbances in biological rhythms. The original BRIAN comprises 18 items divided into four main areas related to circadian rhythm disturbances: sleep, social rhythms, activity, and eating patterns. The total BRIAN scores from the 18 items range from 18 to 72, with higher scores indicating a more severe circadian rhythm disturbance. The Korean version, K-BRIAN, has been adapted and validated for the Korean population to ensure cultural and linguistic appropriateness. K-BRIAN comprises a series of questions that evaluate disruptions in various biological rhythms, including sleep–wake cycles, meal patterns, and other daily activities. Each item on the K-BRIAN is rated on a scale, with higher scores indicating more significant disturbances in biological rhythms. The questionnaire covers five main domains: sleep, activities, socialization, eating patterns, and time types about morningness-eveningness. Participants are asked to reflect on their experiences over a specified period and provide responses that best describe their current state. The K-BRIAN has demonstrated good reliability and validity in the Korean context, making it a valuable tool for researchers and clinicians working with Korean populations. Its utility extends beyond mood disorders, as it can be employed in various neuropsychiatric conditions where biological rhythm disruptions are of concern [21].

### 2.3. Data Analysis

Data analysis was executed using the SPSS software (version 25.0; SPSS et al., Chicago, IL, USA), with a significance threshold set at *p* < 0.05. The initial analysis involved comparing participant demographic and clinical characteristics using means and standard deviations. An independent sample *t*-test assessed the differences between first and recurrent episode groups for the comparative analysis of mood, anxiety, and circadian rhythm scales. Pearson’s correlation analysis was subsequently employed to determine the correlation between mood scales and circadian rhythm-related scales in the whole group and each group separately.

## 3. Results

### 3.1. Demographic Overview

The study encompassed 61 adolescents aged 13–18 years. Among them, 22 were diagnosed with a first episode of depression, while 39 had recurrent episodes. The average age for the first episode group was 15.09 ± 1.44 years, comprising 9 boys (40.9%) and 13 girls (59.1%). The recurrent episode group had an average age of 15.95 ± 1.26 years, with a gender distribution of 9 males (23.1%) and 30 females (76.9%). Detailed demographic data are delineated in Table 1.

### 3.2. Comparative Analysis of Mood, Anxiety, and Circadian Rhythm Scales

The research juxtaposed scales pertinent to depression and anxiety (CDI, STAI, K-MDQ-A) with those related to circadian rhythms (SPAQ, KtCS, K-BRIAN). The comparison between first-time depression patients and those with recurrent episodes revealed no significant score disparities across scales (CDI: First Episode: Mean = 26.18 ± 10.54; Recurrent Episode: Mean = 25.90 ± 10.59; *p* = 0.92. STAI-S: First Episode: Mean = 56.91 ± 12.12; Recurrent Episode: Mean = 57.49 ± 11.93; *p* = 0.86. STAI-T: First Episode: Mean = 60.36 ± 11.63; Recurrent Episode: Mean = 59.09 ± 12.10; *p* = 0.68. K-MDQ-A: First Episode: Mean = 1.50 ± 1.87; Recurrent Episode: Mean = 2.44 ± 2.59; *p* = 0.14. SPAQ-total: First Episode: Mean = 6.59 ± 4.86; Recurrent Episode: Mean = 6.77 ± 5.23; *p* = 0.90. KtCS: First Episode: Mean = 30.32 ± 5.83; Recurrent Episode: Mean = 28.13 ± 7.36; *p* = 0.24.). This suggests that the severity or recurrence of depression episodes might not directly influence the circadian rhythm patterns. Comprehensive results are showcased in Table 2.

### 3.3. Correlation between Mood Scales and Circadian Rhythm-Related Scales in the Entire Group

Pearson’s correlation analysis was performed on mood and anxiety scales, such as the CDI, STAI, and K-MDQ-A, and circadian rhythm-related scales, such as the SPAQ, KtCS, and K-BRIAN. Significant correlations were identified between CDI and SPAQ-weight (r = 0.26, *p* < 0.05), CDI and KtCS (r = −0.48, *p* < 0.01), CDI and K-BRIAN-sleep (r = 0.58, *p* < 0.01), CDI and K-BRIAN-activity (r = 0.64, *p* < 0.01), CDI and K-BRIAN-social (r = 0.71, *p* < 0.01), and CDI and K-BRIAN-eating (r = 0.40, *p* < 0.05). Significant correlations were identified between the STAI-S and SPAQ-energy (r = 0.28, *p* < 0.05), STAI-S and KtCS (r = −0.41, *p* < 0.01), STAI-S and K-BRIAN-sleep (r = 0.59, *p* < 0.01), STAI-S and K-BRIAN-activity (r = 0.56, *p* < 0.01), STAI-S and K-BRIAN-social (r = 0.60, *p* < 0.01), and STAI-S and K-BRIAN-eating (r = 0.32, *p* < 0.05). Significant correlations were identified between STAI-T and SPAQ-energy (r = 0.28, *p* < 0.05), STAI-T and SPAQ-total (r = 0.25, *p* < 0.05), STAI-T and KtCS (r = −0.43, *p* < 0.01), STAI-T and K-BRIAN-sleep (r = 0.59, *p* < 0.01), STAI-T and K-BRIAN-activity (r = 0.63, *p* < 0.01), STAI-T and K-BRIAN-social (r = 0.52, *p* < 0.01), and STAI-T and K-BRIAN-eating (r = 0.41, *p* < 0.01). Finally, a significant correlation was identified between the K-MDQ-A and SPAQ-sleep (r = 0.35, *p* < 0.01) and the K-MDQ-A and SPAQ-total (r = 0.26, *p* < 0.05). The results of the analysis are presented in Table 3.

### 3.4. Group-Specific Correlation Analysis

Pearson’s correlation analysis was performed on mood and anxiety scales, such as CDI, STAI, and K-MDQ-A, and circadian rhythm-related scales, such as SPAQ, KtCS, and K-BRIAN, in each group.

In the first episode group, significant correlations were identified between CDI and SPAQ-mood (r = 0.54, *p* < 0.01), CDI and SPAQ-weight (r = 0.55, *p* < 0.01), CDI and SPAQ-total (r = 0.44, *p* < 0.05), CDI and K-BRIAN-sleep (r = 0.57, *p* < 0.01), CDI and K-BRIAN-activity (r = 0.66, *p* < 0.01), CDI and K-BRIAN-social (r = 0.66, *p* < 0.01), and CDI and K-BRIAN-eating (r = 0.45, *p* < 0.05). Significant correlations were identified between the STAI-S and SPAQ-mood (r = 0.44, *p* < 0.05), STAI-S and K-BRIAN-sleep (r = 0.58, r < 0.01), STAI-S and K-BRIAN-activity (r = 0.58, *p* < 0.01), STAI-S and K-BRIAN-social (r = 0.57, *p* < 0.01), and STAI-S and K-BRIAN-eating (r = 0.49, *p* < 0.05). Significant correlations were identified between the STAI-T and SPAQ-mood (r = 0.46, *p* < 0.05), STAI-T and K-BRIAN-sleep (r = 0.51, *p* < 0.05), STAI-T and K-BRIAN-activity (r = 0.65, *p* < 0.01), STAI-T and K-BRIAN-social (r = 0.51, *p* < 0.05), and STAI-T and K-BRIAN-eating (r = 0.47, *p* < 0.05). Finally, a significant correlation was identified between K-MDQ-A and KtCS (r = −0.43, *p* < 0.05) and K-MDQ-A and K-BRIAN-eating (r = 0.52, *p* < 0.05). The results of the analysis are presented in Table 4.

In the recurrent episode group, a significant correlation was identified between CDI and KtCS (r = −0.62, *p* < 0.01), CDI and K-BRIAN-sleep (r = 0.58, *p* < 0.01), CDI and K-BRIAN-activity (r = 0.64, *p* < 0.01), CDI and K-BRIAN-social (r = 0.75, *p* < 0.01), and CDI and K-BRIAN-eating (r = 0.39, *p* < 0.05). Significant correlations were identified between the STAI-S and KtCS (r = −0.58, *p* < 0.01), STAI-S and K-BRIAN-sleep (r = 0.59, *p* < 0.01), STAI-S and K-BRIAN-activity (r = 0.54, *p* < 0.01), and STAI-S and K-BRIAN-social (r = 0.61, *p* < 0.01). Significant correlations were identified between the STAI-T and KtCS (r = −0.64, *p* < 0.01), STAI-T and K-BRIAN-sleep (r = 0.63, *p* < 0.01), STAI-T and K-BRIAN-activity (r = 0.63, *p* < 0.01), STAI-T and K-BRIAN-social (r = 0.52, *p* < 0.01), and STAI-T and K-BRIAN-eating (r = 0.41, *p* < 0.05). Finally, significant correlations were identified between the K-MDQ-A and SPAQ-sleep (r = 0.39, *p* < 0.05), K-MDQ-A and SPAQ-energy (r = 0.33, *p* < 0.05), and K-MDQ-A and SPAQ-total (r = 0.34, *p* < 0.05). The results of the analysis are presented in Table 4.

## 4. Discussion

Many patients with depressive disorders experience symptoms about sleep behavior and daily rhythmicity. However, it still needs to be made clear which is the cause and which is the effect. The intricate relationship between circadian rhythms and adolescent depression is a burgeoning area of interest in neuropsychiatry. While the interplay between these two entities has been acknowledged, the depth and breadth of their connection remain to be fully elucidated. Historically, severe adolescent depression has been associated with extended circadian rhythm durations, suggesting a potential physiological manifestation of the disorder [22]. Most of the studies showed an association between a late chronotype and depressive symptomatology in adolescents [23]. Our study sought to delve deeper into this relationship, aiming to provide a more comprehensive understanding.

Utilizing a trio of circadian rhythm assessment tools (SPAQ, KtCS, K-BRIAN), our research juxtaposed their findings with established tools that gauge depression, anxiety, and bipolarity. This amalgamation of instruments, spanning from daily fluctuations to broader annual seasonal changes, provided a multi-dimensional lens through which the relationship between time and mood disorders could be viewed. Our findings, which indicate distinct impacts across each temporal dimension, underscore the potential for a multifaceted therapeutic approach targeting different aspects of circadian disruptions.

In examining annual seasonal patterns, our data unveiled an intriguing assumption. The SPAQ-sleep (r = 0.39, *p* < 0.05), SPAQ-energy (r = 0.33, *p* < 0.05), and SPAQ-total (r = 0.34, *p* < 0.05) scores exhibited significant positive correlations with the K-MDQ-A in the recurrent episode group. This observation raises a tantalizing hypothesis: adolescents with depression who manifest pronounced circadian rhythm disruptions, especially in seasonality, might harbor an increased susceptibility to bipolar tendencies. This potential link between seasonality and bipolarity could have profound implications for early detection and intervention strategies. From a clinical vantage point, the trajectory of certain adolescents is of particular interest. Some individuals, initially presenting with depressive episodes, might eventually manifest manic episodes, leading to a bipolar disorder diagnosis. Predicting this transition is academically intriguing and holds immense clinical significance. While numerous studies have delved into genetic and environmental risk factors for bipolar disorder [24,25], our results introduce a novel perspective. They highlight the intricate link between recurrent depressive episodes, pronounced seasonality patterns, and heightened bipolar tendencies. Furthermore, as depression evolves into a chronic condition, there is a discernible shift towards evening-type behaviors, accompanied by more pronounced circadian rhythm disruptions.

Our exploration into daily circadian rhythms, as assessed by KtCS and K-BRIAN, yielded equally compelling insights. A clear trend emerged, wherein as the severity of depressive symptoms intensified, there was a discernible shift towards evening-type behaviors. This nocturnal inclination, characterized by delayed bedtimes and wake times, resonates with findings from adult populations, suggesting a potential universal trait linking evening tendencies with atypical depression across age groups [26,27].

The K-BRIAN’s comprehensive approach, which evaluates disruptions across four daily life domains, added depth to our analysis. A clear pattern emerged, suggesting a symbiotic relationship between emotional well-being and biological rhythms. As negative emotions intensified, so did biological rhythm disturbances, underscoring their intertwined nature.

Our study’s comparative analysis between first and recurrent episode groups offered unique insights. While the data did not yield stark differences in circadian rhythm metrics, the intricate correlations between circadian rhythm variables were especially pronounced in the recurrent episode group. This observation suggests that as the frequency of depressive episodes increases, the relationship between seasonality and bipolar disorder risk becomes more complex.

In conclusion, our findings paint a multifaceted picture of the relationship between circadian rhythms and adolescent depression. They underscore the potential link between pronounced seasonality patterns and elevated bipolar tendencies in this demographic. As depression’s grip tightens, there is a tangible shift towards evening-type behaviors, with more pronounced biological rhythm disturbances. This association seems to intensify with recurrent depressive episodes.

While our study provides valuable insights, it is crucial to recognize its limitations, notably the small sample size and cross-sectional study design. In addition, future studies should further evaluate and control for other variables that may affect the circadian cycle, such as nighttime use of electronic devices, the presence of other coexisting medical conditions, and irregular sleep patterns. However, we have the advantage of having a more homogeneous group of patients who were accurately diagnosed at a single study site using a rigorous semi-structured clinical assessment interview tool. Its focused approach and differentiation set it apart, offering a fresh perspective on a multifaceted issue. Future research endeavors, encompassing larger cohorts and diverse populations, could further illuminate these findings, potentially leading to therapeutic interventions tailored to individual circadian profiles.

Personalized medicine means interventions tailored to individuals’ unique characteristics at the molecular, physiological, and behavioral levels and environmental exposures to address their specific disease manifestations effectively [28]. As we better understand the relationship between depression and circadian rhythms, we may be able to tailor our understanding of depression to the individualized nature of circadian rhythms in each person. This understanding goes hand in hand with personalized medicine, which allows for an individualized approach. As personalized medicine continues to evolve, integrating insights from circadian biology with clinical psychiatry can revolutionize how we approach, diagnose, and treat MDD.

## 5. Conclusions

This study elucidates a complex relationship between circadian rhythms and adolescent depression, highlighting a notable link between distinct seasonality patterns and increased bipolar tendencies in this demographic. A clear shift towards evening-type behaviors and more pronounced biological rhythm disturbances are observed as depression severity escalates, with these associations intensifying alongside recurrent depressive episodes. While providing valuable insights, it is critical to acknowledge the limitations of this study, particularly its modest sample size. Despite these constraints, the study offers a new perspective on a multifaceted issue. Future research involving larger and more diverse cohorts is essential to explore these findings further and potentially guide the development of therapeutic interventions tailored to individual circadian profiles.

## Figures and Tables

**Table 1:** Basic demographic data.

	Episode		Total
Clinical Characteristics	First (22)	Recurrent (39)	61
Age	15.09 ± 1.44 (years)	15.95 ± 1.26 (years)	
Sex	Total number (percentage)		
Male	9 (40.9%)	9 (23.1%)	18
Female	13 (59.1%)	30 (76.9%)	43
Total	22	39	61

**Table 2:** Comparisons of scale scores between the first episode and recurrent episode group.

Episode	First Episode			Recurrent Episode			
	Number (22)	Mean	SD	Number (39)	Mean	SD	*p* Value
**CDI**		26.18	10.54		25.90	10.59	0.92
**STAI-S**		56.91	12.12		57.49	11.93	0.86
**STAI-T**		60.36	11.63		59.09	12.10	0.68
**K-MDQ-A**		1.50	1.87		2.44	2.59	0.14
**SPAQ-sleep**		1.18	1.05		1.44	1.23	0.42
**SPAQ-social**		1.09	1.19		0.77	0.93	0.25
**SPAQ-mood**		1.45	1.22		1.41	1.39	0.90
**SPAQ-weight**		0.77	1.07		1.03	1.11	0.39
**SPAQ-energy**		1.32	1.17		1.23	1.33	0.80
**SPAQ-appetite**		0.77	1.15		0.90	1.07	0.67
**SPAQ-total**		6.59	4.86		6.77	5.23	0.90
**KtCS**		30.32	5.83		28.13	7.36	0.24
**K-BRIAN-sleep**		13.45	3.75		13.51	4.03	0.96
**K-BRIAN-activity**		11.14	3.63		11.77	3.89	0.54
**K-BRIAN-social**		9.18	3.00		9.15	2.93	0.97
**K-BRIAN-eating**		8.77	3.02		9.82	3.93	0.28

Abbreviations: CDI = the Children’s Depression Inventory; STAI = the State-Trait Anxiety Inventory; MDQ = The Mood Disorder Questionnaire; SPAQ = the Seasonal Pattern Assessment Questionnaire; KtCS = the Korean Translation of Composite Scale to Measure Morningness-Eveningness; K-BRIAN = the Korean version of the Biological Rhythms Interview of Assessment in Neuropsychiatry; SD = standard deviation.

**Table 3:** Correlations between mood and circadian rhythm scale scores in the whole group.

	CDI	STAI-S	STAI-T	K-MDQ-A
SPAQ-sleep	0.11	0.15	0.21	0.35 **
SPAQ-social	−0.04	0.09	0.02	0.05
SPAQ-mood	0.17	0.16	0.20	0.13
SPAQ-weight	0.26 *	0.17	0.18	0.16
SPAQ-energy	0.14	0.28 *	0.28 *	0.24
SPAQ-appetite	0.12	0.14	0.18	0.21
SPAQ-total	0.18	0.23	0.25 *	0.26 *
KtCS	−0.48 **	−0.41 **	−0.43 **	−0.07
K-BRIAN-sleep	0.58 **	0.59 **	0.59 **	0.05
K-BRIAN-activity	0.64 **	0.56 **	0.63 **	0.02
K-BRIAN-social	0.71 **	0.60 **	0.52 **	−0.05
K-BRIAN-eating	0.40 *	0.32 *	0.41 **	0.10

* *p* < 0.05, ** *p* < 0.01. Abbreviations: SPAQ = the Seasonal Pattern Assessment Questionnaire; KtCS = the Korean Translation of Composite Scale to Measure Morningness-Eveningness; K-BRIAN = the Korean version of the Biological Rhythms Interview of Assessment in Neuropsychiatry.

**Table 4:** Correlations between mood and circadian rhythm scale scores in each group.

		CDI	STAI-S	STAI-T	K-MDQ-A
**First episode**	SPAQ-sleep	0.30	0.35	0.39	0.17
	SPAQ-social	−0.10	0.07	−0.04	−0.19
	SPAQ-mood	0.54 **	0.44 *	0.46 *	0.06
	SPAQ-weight	0.55 **	0.26	0.28	0.06
	SPAQ-energy	0.32	0.42	0.39	0.01
	SPAQ-appetite	0.28	0.19	0.26	0.19
	SPAQ-total	0.44 *	0.41	0.41	0.07
	KtCS	−0.19	−0.06	0.01	−0.43 *
	K-BRIAN-sleep	0.57 **	0.58 **	0.51 *	0.05
	K-BRIAN-activity	0.66 **	0.58 **	0.65 **	0.10
	K-BRIAN-social	0.66 **	0.57 **	0.51 *	0.30
	K-BRIAN-eating	0.45 *	0.49 *	0.47 *	0.52 *
**Recurrent episode**	SPAQ-sleep	0.02	0.05	0.13	0.39 *
	SPAQ-social	0.00	0.11	0.04	0.23
	SPAQ-mood	−0.02	0.02	0.08	0.16
	SPAQ-weight	0.11	0.12	0.15	0.17
	SPAQ-energy	0.05	0.22	0.23	0.33 *
	SPAQ-appetite	0.02	0.11	0.14	0.22
	SPAQ-total	0.04	0.14	0.18	0.34 *
	KtCS	−0.62 **	−0.58 **	−0.64 **	0.08
	K-BRIAN-sleep	0.58 **	0.59 **	0.63 **	0.05
	K-BRIAN-activity	0.64 **	0.54 **	0.63 **	−0.02
	K-BRIAN-social	0.75 **	0.61 **	0.52 **	−0.19
	K-BRIAN-eating	0.39 *	0.25	0.41 *	−0.07

* *p* < 0.05, ** *p* < 0.01. Abbreviations: SPAQ = the Seasonal Pattern Assessment Questionnaire; KtCS = the Korean Translation of Composite Scale to Measure Morningness-Eveningness; K-BRIAN = the Korean version of the Biological Rhythms Interview of Assessment in Neuropsychiatry.

## Data Availability

The data presented in this study are available on request from the corresponding author. The data are not publicly available due to privacy.
